# Role of Suppressor of Cytokine Signaling-1 In Murine Atherosclerosis

**DOI:** 10.1371/journal.pone.0051608

**Published:** 2012-12-27

**Authors:** Christina Grothusen, Harald Schuett, Anja Hillmer, Stefan Lumpe, Karsten Grote, Matthias Ballmaier, Andre Bleich, Silke Glage, Uwe J. F. Tietge, Maren Luchtefeld, Bernhard Schieffer

**Affiliations:** 1 Department of Cardiology and Angiology, Hannover Medical School, Hannover, Germany; 2 Department of Pediatric Haematology and Oncology, Hannover Medical School, Hannover, Germany; 3 Institute for Laboratory Animal Science, Hannover Medical School, Hannover, Germany; 4 Department of Pediatrics, Center for Liver, Digestive and Metabolic Diseases, University Medical Center Groningen, University of Groningen, The Netherlands; University of Freiburg, Germany

## Abstract

**Background:**

While the impact of inflammation as the substantial driving force of atherosclerosis has been investigated in detail throughout the years, the influence of negative regulators of pro-atherogenic pathways on plaque development has remained largely unknown. Suppressor of cytokine signaling (SOCS)-1 potently restricts transduction of various inflammatory signals and, thereby modulates T-cell development, macrophage activation and dendritic cell maturation. Its role in atherogenesis, however has not been elucidated so far.

**Methods and Results:**

Loss of SOCS-1 in the low-density lipoprotein receptor deficient murine model of atherosclerosis resulted in a complex, systemic and ultimately lethal inflammation with increased generation of Ly-6C^hi^ monocytes and activated macrophages. Even short-term exposure of these mice to high-cholesterol dieting caused enhanced atherosclerotic plaque development with accumulation of M1 macrophages, Ly-6C positive cells and neutrophils.

**Conclusion:**

Our data not only imply that SOCS-1 is athero-protective but also emphasize the fundamental, regulatory importance of SOCS-1 in inflammation-prone organisms.

## Introduction

Atherosclerotic plaque growth and rupture are considered as the underlying cause of myocardial infarction, sudden cardiac death and stroke. While the fundamental contribution of inflammation to atherogenesis has already been extensively studied, approaches to determine the impact of inhibitors of inflammatory signaling pathways have remained few so far. [1] Suppressor of cytokine signalling (SOCS)-1 belongs to a family of 8 intracellular proteins and restricts type I Interferon and Interferon(IFN)-γ receptor activation. Thereby, SOCS-1 acts as a classical negative feedback loop regulator of the Janus kinase and – signal transducer and activator of transcription (JAK-STAT) pathway. [2] Furthermore, SOCS-1 restricts toll-like receptor activation and modulates nuclear factor (NF)-κβ dependent transcription by binding to and induction of degradation of the p65 subunit. Thereby, SOCS-1 controls innate and adaptive inflammatory cell behaviour, including macrophages, granulocytes, dendritic cells and T-cells in response to a diverse set of pro-atherogenic cytokines, e.g. interleukin-6, tumor necrosis factor (TNF)-α, and IFN-γ. [3], [4].

SOCS-1 as well as SOCS-3 expression has recently been demonstrated in murine and human atherosclerotic lesions. In addition, in vitro studies suggested an anti-inflammatory and potentially athero-protective effect of these molecules in vascular cells, e.g. monocytes, endothelial and smooth muscle cells. [5] While T-cell specific loss of SOCS-3 resulted in reduced plaque development, the role of SOCS-1 in atherosclerosis has not been determined yet. [5], [6] Therefore, we investigated the impact of a systemic deficiency of SOCS-1 on atherogenesis in the established, murine low-density lipoprotein receptor (LDLR) model of atherosclerosis. We hypothesised that loss of SOCS-1 in this setting would result in advanced plaque development. In fact, our data confirm athero-protective features of this molecule but also underline the crucial, gate keeping function of SOCS-1 in inflammation.

## Materials and Methods

### Animals

Socs-1, Rag-2 deficient animals (B6;129/Sv-Socs1*^tm1jni^*-Rag2*^tm1^*, kind gift from J. Ihle, St. Jude Childrens hospital, Memphis, USA) were crossed with low density lipoprotein receptor (Ldlr)-deficient mice (B6.129S7-Ldlr*^tm1Her^*/J, Jackson Laboratories, Maine, USA) to obtain triple knockout (KO) mice (Ldlr^−/−^;Rag-2^−/−^;Socs-1^−/−^, hereafter abbreviated as Socs-1^−/−^triple-KO). Ldlr^−/−^;Rag-2^−/−^;Socs-1^+/+^ (Ldlr^−/−^;Rag-2^−/−^) or Ldlr^−/−^;Rag-2^+/+^;Socs-1^+/+^ (Ldlr^−/−^) mice served as controls if not indicated otherwise. Experiments were performed with male and female mice. No sex-related differences were observed (data not shown). All mice were barrier maintained; routine microbiological monitoring according to FELASA recommendations did not reveal any evidence of infection with common murine pathogens. This study was conducted in accordance with the German animal protection law and with the European Communities Council Directive 86/609/EEC for the protection of animals used for experimental purposes. All experiments were approved by the Local Institutional Animal Care and Research Advisory Committee (Institute for Laboratory Animal Science, Hannover Medical School) and permitted by the local government (Niedersächsisches Landesamt für Verbraucherschutz und Lebensmittelsicherheit). The general health status of these animals was monitored by daily controls of physical appearance and determination of an activity score (1 = lazy, moving slowly; 2 = intermediate; 3 = active moving or searching for food) as well as weekly controls of body weight. Animals were killed when signs of weight loss or stagnation combined with impaired activity were observed. At four weeks of age, Ldlr^−/−^, Ldlr^−/−^;Rag-2^−/−^ and Socs-1^−/−^triple-KO mice were subjected to a high-cholesterol diet (HCD, (D12108, Research Diets; 1.25% Cholesterol without cholate, Brogaarden, Denmark) for 4 weeks or remained on standard chow diet (CD, Altromin® 1314, Lage, Germany) containing 22.5% protein, 5.0% fat and 4.5% fibre. Subsequently, mice were euthanized, organs removed and subjected to further analyses.

### Genotyping

The genotype of each mouse was verified by PCR on genomic DNA (tail tip digest) using the following primers: Ldlr sense: 5′-ACC CCA AGA CGT GCT CCC AGG ATG A-3′, Ldlr anti-sense 5′-CGC AGT GCT CCT CAT CTG ACT TGT-3′, Rag-2-1∶5′-GGG AGG ACA CTC CAC TTG CCA GTA-3′, Rag-2-2∶5′-AGT CAG GAG TCT CCA TCT CAC TAA-3′, Rag-2 neomcyin: 5′-CGG CCG GAG AAC CTG CGT GCA A-3′, Socs-1 sense: 5′-CAG GCA CCC ACT CCT GGC CTT-3′, Socs-1 anti-sense-1∶5′-TGG CCA TTC GGC CTG GC CTT-3′, Socs-1 anti-sense-2∶5′-GCC TTC TTG ACG AGT TCT TCT G-3′.

### Aortic Lipid Deposition

For the analysis of aortic lipid depositions, aortas were prepared en face and stained with Oil Red O solution as previously described. [7] Percentage of Oil Red O positive area was calculated via computer-assisted image quantification (Leica Qwin 500, Leica, Heidelberg, Germany).

### Atherosclerotic Plaque Analysis

To analyse atherosclerotic plaque area and composition, aortic roots were embedded in Tissue-Tek® O.C.T™ and kept at −80°C until sectioning. Within the aortic root, lesion area was analyzed in cross sections obtained at the level of all three leaflets of the aortic valve after staining with Oil Red O solution as previously described. [8] Serial cross sections (5 µm) were collected, 6–9 sections/animals were analysed via computer-assisted image quantification (Leica Qwin 500, Leica, Heidelberg, Germany). Neutrophils were identified using a rat anti-mouse Ly-6G antibody (clone IA8, BD Bioscience, San Jose, USA). Monocytes/macrophages were detected with a rat anti-mouse MOMA-2 antibody (Acris, Herford, Germany), the biotinylated secondary antibody (rabbit anti-rat) was visualized by ABC reagent (Vector Laboratories, Burlingame, USA) and the AEC-Chromogen (DAKO, Glostrup, Denmark) according to the manufacturer’s protocol. Sections were counterstained with hematoxylin (Carl Roth, Karlsruhe, Germany). For visualisation of Ly-6C/MOMA-2 double positive cells, sections were incubated with biotinylated anti-mouse Ly-6C antibody (AL-21, BD Pharmingen, Franklin Lakes, USA) and rat anti-mouse MOMA-2 followed by visualization using streptavidin-Fitc and anti-rat Alexa-549 (Invitrogen, Molecular Probes, Darmstadt, Germany). For staining of CD68/iNOS and CD68/CD206 double-positive cells, we followed a protocol published by Salagianni et al. [9] In short, sections were incubated with rat anti-mouse CD68 (clone FA-11, AbD serotec, Oxford, UK) and rabbit anti-mouse iNOS (AbCam, Cambridge, UK) or rabbit anti-mouse CD206 (clone MR5D3, AbD serotec, Oxford, UK) or respective isotype controls followed by incubation with anti-rat Alexa-549 or anti-rabbit Alexa-488 (both Invitrogen, Molecular Probes, Darmstadt, Germany). Nuclei were counterstained with DAPI (Invitrogen, Molecular Probes, Darmstadt, Germany).

### Histological Analysis

Paraffin embedded, serial sections from various organs were subjected to hematoxylin/eosin (HE) staining. In short, nuclei were stained with alum hematoxylin and differentiated with acid alcohol followed by staining with eosin solution. For Sirius Red staining, we followed a protocol published elsewhere. [10] In short, sections were stained with Sirius Red solution (0.1% Sirius Red in saturated picric acid solution). Section analysis was carried out using polarization microscopy.

### Plasma Analysis

Systemic Tumor necrosis factor (TNF)-α, Interleukin (IL)-6 and MCP (monocyte chemotactic protein)-1 levels were measured using ELISA (all from R&D Systems, Minneapolis, USA) following the manufacturers protocol.

### Lipoprotein Fraction Analysis

The concentration of total cholesterol and triglycerides in plasma was determined using enzymatic colorimetric assays (Roche Diagnostics, Grenzach, Germany). The distribution of cholesterol over the different lipoprotein subclasses in plasma was determined in two different ways. Fast protein liquid chromatography (FPLC) of 100 µL of pooled plasma samples from the respective experimental groups using a Superose 6 column (LKB Biotechnology, Uppsala, Sweden) was carried out as described previously. [11] In addition, sequential tabletop ultracentrifugation was performed to separate VLDL, LDL and HDL as published. [11] Total cholesterol content in the respective fractions was determined using enzymatic colorimetric assays (Roche Diagnostics, Grenzach, Germany).

### LDL-Isolation and Peroxidation

For in-vitro experiments, LDL was isolated from human plasma by sequential gradient ultracentrifugation. [12] LDL fraction was dialyzed at 4°C against phosphate buffer (140 mM NaCl, 1.9 mM NaH2HPO4, 8.1 mM). Protein concentration was determined by the Bradford method. For peroxidation, native (n)LDL (100 µg/mL) was incubated with CuSO_4_ (10 µmol/L) in cell culture medium for 24 hrs. LDL-peroxidation was stopped with EDTA (5 mmol/L) and butylhydroxytoluol (20 µmol/L). LDL-peroxidation was analyzed by detection of conjugated diene formation by measuring UV absorbance at 234 nm. [13] Additionally malondialdehyde as lipid peroxidation product was measured using the thiobarbituric acid-reactive assay (TBARS assay kit, Oxitech, Buffalo, USA).

### Generation of Bone-Marrow Derived Macrophages

To generate macrophages, bone marrow cells (BMC) were isolated and cultivated in RPMI 1640 (GIBCO/Invitrogen Darmstadt, Germany), supplemented with 10% fetal calf serum (FCS) +1% penicillin/streptomycin and 20% L929 conditioned medium for 7 days as previously described. [14].

### In Vitro Foam Cell Formation

BMC were differentiated into bone-marrow derived macrophages (BMDM) as described above. In-vitro foam cell formation was induced by incubating BMDM with 25 µg/mL oxidized (ox)LDL and nLDL for 4 hrs. After stimulation, cells were washed twice with PBS, fixated in 3.7% paraformaldehyde followed by staining of intracellular lipids with Oil red O for 30 min. The percentage of Oil red O positive stained cells was quantified by randomly choosing 5 microscopic fields using computer-assisted image analysis (Axiovert 200 M). [15].

### Scavenger Receptor A and CD36 Expression

BMDM were stimulated with oxLDL or nLDL for 24 hrs followed by double-staining of scavenger receptor (SR) A (anti-mouse CD204-Alexa-Fluor 647, AbD Serotec, Oxford, UK) and CD36 (anti-mouse CD36-PE, eBioscience, San Diego, USA) or the respective isotype controls. Unspecific binding of antibodies to Fc receptors was reduced by pre-incubation of BMDM with CD16/32 (anti-mouse CD16/CD32, eBioscience). Expression was analysed by flow cytometry (FACSCalibur, Becton, Dickinson and Company, Franklin Lakes, USA).

### Cell Sorting

Cells were harvested from bone marrow, peripheral blood and spleens as described by Swirski et al. [16] BMCs from both tibias and femurs were harvested. Peripheral blood was drawn via cardiac puncture and collected into EDTA tubes. Mononuclear cells were purified by density centrifugation. Differential blood counts were obtained using the Vet abc Animal Blood Counter (scil animal care company GmbH, Viernheim, Germany). Spleens were removed and filtered through a nylon mesh (BD Bioscience). The cell suspensions were centrifuged, red blood cells were lysed with NH_4_Cl (0.155 M), and the resulting single-cell suspensions were washed with PBS supplemented with 0.2% EDTA and 2% FCS. Cells were incubated with a cocktail of monoclonal antibodies against T cells (anti CD90-PE, clone 53–2.1, BD Bioscience), B cells (anti CD45R-PE, B220, clone RA3-6B2, eBioscience), NK cells (anti CD49b-PE, clone DX5, eBioscience and NK1.1-PE, clone PK136, BD Bioscience), granulocytes (anti Ly-6G–PE, clone 1A8, BD Bioscience), myeloid cells (anti CD11b-APC, clone M1/70, eBioscience) and monocyte subsets (anti Ly-6C–FITC, clone AL-21, BD Bioscience). Monocytes were identified as CD11b^hi^ (CD90, B220, CD49b, NK1.1, Ly-6G)^lo^cells. Macrophage marker F4/80-PE (ebioscience), the MHC class II anti-I-Ab-PE (BD Bioscience), and CD11c-PE (eBioscience) mAbs were additionally used to identify CD11b^hi^ (CD90, B220, CD49b, NK1.1, Ly-6G, F4/80, CD11c, I-AB)^lo^ cells in spleens. Monocyte subsets were further identified as either Ly-6C^hi^ or Ly-6C^lo^.

### Statistical Analysis

Data are presented as mean and standard error of mean (SEM) or standard deviation (SD). Statistical analyses were performed using SigmaStat 3.0 (SyStat, San Jose, USA). Comparisons between two groups were performed by Student's t-test assuming two-tailed distribution and equal variances. One-way ANOVA was used for the comparisons between three groups. For unequal group sizes with failed normality tests, Rank-based ANOVA was used and data presented as median with 25^th^ and 75^th^ percentile. P values <0.05 were considered statistically significant.

## Results

### Deficiency of SOCS-1 in Atherosclerosis-prone Mice Results in a Lethal, Systemic Inflammation

To assess the impact of SOCS-1 deficiency on atherogenesis, Ldlr^+/+^;Rag-2^−/−^;Socs-1^−/−^ (Rag-2^−/−^;Socs-1^−/−^) mice were crossed with Ldlr^−/−^;Rag-2^+/+^;Socs-1^+/+^ (Ldlr^−/−^) mice to generate Ldlr^−/−^;Rag-2^−/−^;Socs-1^−/−^ (Socs-1^−/−^ triple-KO) mice. The necessity of combining a systemic lack of SOCS-1 with a RAG-2 deficiency is due to the fact that Socs-1^−/−^ mice suffer from a severe hypersensitivity towards IFN-γ signaling that leads to death within 3 weeks after birth. Lack of RAG-2, however results in depletion of lymphocytes, a major source for IFN-γ. Accordingly, Rag-2^−/−^;Socs-1^−/−^ mice have been demonstrated to survive until adulthood. [17].

**Figure 1:**
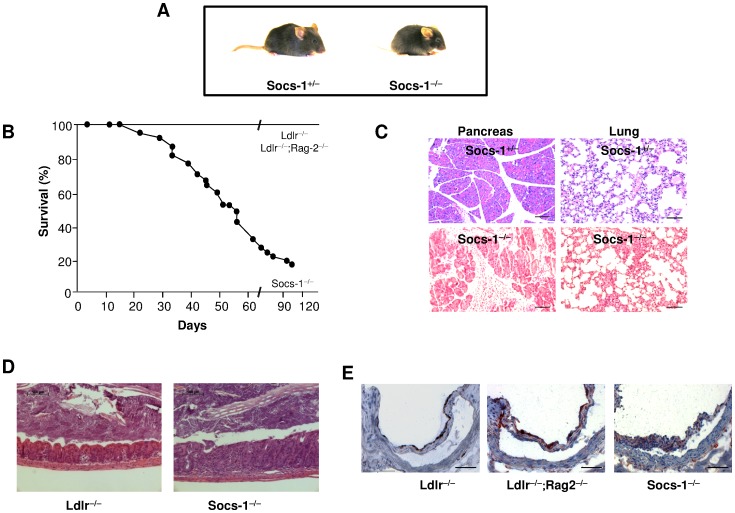
Impact of SOCS-1 deficiency on inflammation and survival. A. Representative picture showing appearance of Socs-1^−/−^triple-KO mice compared to Ldlr^−/−^;Rag-2^−/−^;Socs-1^+/−^ (Socs-1^+/−^) mice. **B.** Survival of Socs-1^−/−^ triple-KO mice (N = 22) compared to Ldlr^−/−^ and Ldlr^−/−^;Rag-2^−/−^ animals (N = 40) demonstrating increased mortality of Socs-1^−/−^triple-KO mice **C.** Representative pictures (HE) of pancreas and lung of Socs-1^−/−^ triple-KO mice compared to Socs-1^+/−^ animals demonstrating increased, diffuse infiltration of mixed inflammatory cells (lymphocyte-like cells, and neutrophils) in both organs (scale bar 100 µm). **D.** Representative pictures (HE) of colon showing typhlitis in Socs-1^−/−^ triple-KO mice compared to Ldlr^−/−^animals. **E.** Representative pictures of aortic roots derived from Ldlr^−/−^, Ldlr^−/−^;Rag-2^−/−^ and Socs-1^−/−^ triple-KO mice showing comparable macrophage infiltration without formation of atherosclerotic plaques under chow diet (scale bar: 50 µm).

Socs-1^−/−^ triple-KO mice exhibited lower weight gain and growth retardation (table 1 and figure 1A) and died within 120 days after birth (figure 1B). Every other documented genotype appeared to be healthy including heterozygous Ldlr^−/−^;Rag-2^−/−^;Socs-1^+/−^ mice. Histological analysis by HE staining of organs derived from Socs-1^−/−^triple-KO mice showed a systemic inflammation with multi-organ infiltration of mixed inflammatory cells (figure 1C) as well as signs of colitis (figure 1D). Atherosclerotic plaque development in aortic roots was not detected under CD (figure 1E). As demonstrated by differential blood counts, Ldlr^−/−^;Rag-2^−/−^ and Socs-1^−/−^triple-KO mice displayed a significant leucopenia that was compensated in Socs-1^−/−^triple-KO mice by granulocytosis, resulting in similar total circulating leukocyte numbers compared to Ldlr^−/−^ mice. Granulocytosis was mainly due to neutrophilia (table 2). However, while Geissmann et al reported that RAG-2 dependent lymphopenia may be compensated by monocytosis, RAG-2 deficiency in combination with lack of the LDLR did not affect monocyte proportions. [18] Apart from the severely impaired life span, generation of Socs-1^−/−^ triple-KO mice resulted in a phenotype resembling characteristics of both, Rag-2^−/−^;Socs-1^−/−^ and Socs-1^−/−^ animals. [19] Ldlr^−/−^;Rag-2^−/−^ mice, though were indistinguishable from Ldlr^−/−^ animals.

**Table 1:** Body Weight of Mice on Chow and High-Cholesterol Diet.

Genotype	CD(g)	HCD(g)
**Ldlr** ^−/−^	19.35±5.37	22.52±3.79
**Ldlr** ^−/−^;**Rag-2** ^−/−^	16.66±3.20	22.83±−3.77
**Socs-1** ^−/−^	10.61±3.25*	17.74±2.62*#

**Weight of mice on chow (CD) and after high-cholesterol diet (HCD).** Age-matched mice were weighted after 4 weeks of CD or HCD. Socs-1^−/−^ triple KO mice (Socs-1^−/−^) on CD and HCD demonstrated a significantly lower weight compared to Ldlr^−/−^ and Ldlr^−/−^;Rag-2^−/−^ mice. However, Socs-1^−/−^triple-KO mice displayed a significant weight gain after 4 weeks of HCD compared to Socs-1^−/−^triple-KO mice on CD. Data represent mean±SD, *p<0.05 vs Ldlr^−/−^ and Ldlr^−/−^;Rag-2^−/−^ mice, #p<0.05 vs Socs-1^−/−^ triple-KO mice on CD, 13–21 mice/group.

**Table 2:** Blood Count on Chow and High-Cholesterol Diet.

	Total Circulating Leukocytes x10^6^/ml [25th/75th percentiles]		Leukocytes corr. for Granulocytes x10^6^/ml [25th/75th percentiles]		Total Circulating Granulocytes x10^6^/ml [25th/75th percentiles]		Total Circulating Neutrophils x10^6^/ml [25^th^/75^th^ percentiles]	
	CD	HCD	CD	HCD	CD	HCD	CD	HCD
**Ldlr** ^−/−^	1.60 [1.4/1.8]	2.00 [1.6/2.5]	1.05 [0.8/1.2]	1.20 [1.0/1.4]	0.50 [0.5/0.7]	0.80 [0.6/0.9]	0.40 [0.4/0.5]	0.70 [0.5/0.6]
**Ldlr** ^−/−^; **Rag-2** ^−/−^	0.70 [0.6/1.2]#	1.10 [0.9/1.3]	0.00 [0.0/0.0] *	0.30 [0.2/0.3] *	1.00 [0.9/1.0]	0.95 [0.9/1.1]	0.45 [0.0/1.0]	0.80 [0.7/0.9]
**Socs-1** ^−/−^	2.40 [1.9/2.9]	1.5 [1.4/2.9]	0.30 [0.2/0.3] *	0.20 [0.1/0.3] *	2.30 [1.7/2.8]*	1.45 [1.2/2.6]*	1.40 [0.4/2.5]	1.10 [1.0/1.5]

**Blood count on chow (CD) and high-cholesterol diet (HCD).** Whole blood samples of all three genotypes were taken from age-matched mice after 4 weeks of CD or HCD. Differential blood counts were obtained using an automated blood counter. Ldlr^−/−^;Rag-2^−/−^ and Socs-1^−/−^triple-KO mice (Socs-1^−/−^) showed a significant leucopenia after correction for granulocytes compared to Ldlr^−/−^ mice on CD and HCD. Leucopenia in Socs-1^−/−^ triple-KO mice was compensated by a significant granulocytosis under both conditions compared to Ldlr^−/−^ mice. Granulocytes mainly consisted of neutrophils. Data represent median with 25^th^ and 75^th^ percentiles, #p<0.05 vs Ldlr^−/−^ and Socs-1^−/−^ triple-KO mice, *p<0.05 vs Ldlr^−/−^ mice, N = 13–21 mice/group.

### Systemic Deficiency of SOCS-1 Accelerates Early Atherogenesis

To investigate the impact of SOCS-1 on early atherogenesis, we fed Ldlr^−/−^; Ldlr^−/−^;Rag-2^−/−^ and Socs-1^−/−^ triple-KO mice a high-cholesterol diet (HCD) for 4 weeks. To contain any possibly confounding impact of deteriorating organ function in Socs-1^−/^triple-KO mice, HCD was started directly after weaning at the age of 30–40 days.

**Figure 2:**
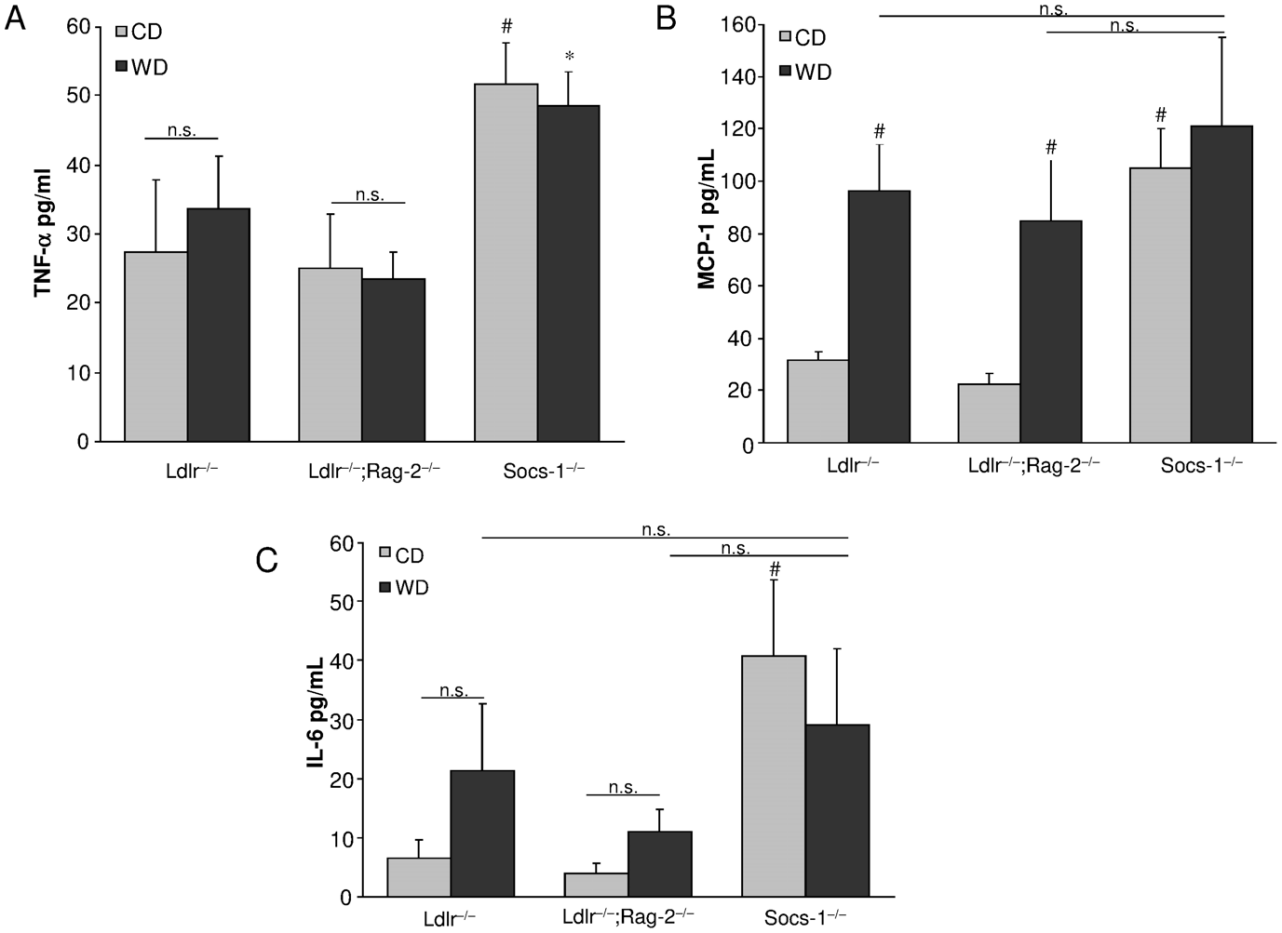
SOCS-1 deficiency results in enhanced levels of pro-inflammatory cytokines. A. Socs-1^−/−^ triple-KO mice (Socs-1^−/−^) showed significantly enhanced circulating TNF-α levels after 4 weeks of CD and HCD while no change was found in Ldlr^−/−^ and Ldlr^−/−^;Rag-2^−/−^ mice. **B and C.** Socs-1^−/−^triple-KO mice (Socs-1^−/−^) showed significantly enhanced levels of MCP-1 (**B**) and IL-6 (**C**) on CD while 4 weeks of HCD did not induce an additional increase in these mice. MCP-1 and IL-6 levels were significantly increased in Ldlr^−/−^ and Ldlr^−/−^;Rag-2^−/−^mice on HCD. *p<0.05 vs Ldlr^−/−^ and Ldlr^−/−^;Rag-2^−/−^ on HCD, #p<0.05 vs Ldlr^−/−^ and Ldlr^−/−^;Rag-2^−/−^ on CD, N = 7 samples/group.

Despite a significant weight gain, Socs-1^−/−^ triple-KO mice remained lighter compared to the other genotypes after 4 weeks of HCD (table 1). Lipoprotein fractions did not differ between Socs-1^−/−^ triple-KO and Ldlr^−/−^ mice. However, Ldlr^−/−^;Rag-2^−/−^ mice displayed decreased total cholesterol levels caused by a lower LDL- and VLDL-cholesterol fraction (table 3). Analysis of plasma samples revealed significantly increased circulating monocyte chemotactic protein (MCP)-1 and interleukin (IL)-6 levels in Ldlr^−/−^ and Ldlr^−/−^;Rag-2^−/−^ mice after HCD while tumor necrosis factor (TNF)-α levels remained unchanged. Socs-1^−/−^triple-KO mice however showed increased levels of all three chemokines/cytokines already under CD while 4 weeks of HCD did not induce any further increase (figure 2A–C).

**Table 3:** Cholesterol Levels after 4 Weeks of High-Cholesterol Diet.

Cholesterol levels (mg/dl)	Ldlr^−/−^	Ldlr^−/−^;Rag-2^−/−^	Socs-1^−/−^triple-KO
**TC**	1108	702*	1018
**LDL**	331	210*	296
**VLDL**	619	351*	515
**HDL**	155	142	161

**Cholesterol levels after 4 weeks of high-cholesterol diet (HCD).** No differences in lipoprotein fractions between age-matched Ldlr^−/−^ and Socs-1^−/−^triple-KO mice were observed after 4 weeks of HCD. Total cholesterol levels were significantly lower in Ldlr^−/−^;Rag-2^−/−^ mice compared to the other genotypes due to lower LDL and VLDL lipoprotein fractions. *p<0.05 vs Ldlr^−/−^ and Socs-1^−/−^triple-KO mice, data are mean ± SD, N = 7–10 mice/group.

**Figure 3:**
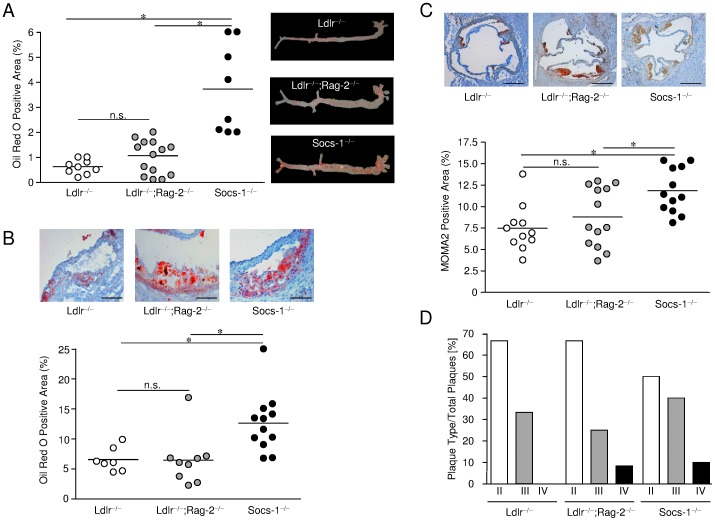
SOCS-1 deficiency results in enhanced atherosclerotic plaque formation. A. Aortas derived from Socs-1^−/−^ triple-KO mice (Socs-1^−/−^) showed significantly enhanced lipid depositions after en face preparation and staining with Oil Red O after 4 weeks of HCD. **B.** Aortic roots of Socs-1^−/−^ triple-KO mice also displayed increased lipid depositions after staining with Oil Red O. Horizontal bars represent mean, *p<0.05 vs Ldlr^−/−^ and Ldlr^−/−^;Rag-2^−/−^ (scale bar: 50 µm). Each dot indicates results for an individual animal. **C.** Aortic roots derived from Socs-1^−/−^ triple-KO (Socs-1^−/−^) mice showed a significantly enhanced macrophage content after staining with MOMA-2 compared to Ldlr^−/−^;Rag-2^−/−^ and Ldlr^−/−^ mice after 4 weeks of HCD. Horizontal bars are mean, *p<0.05 vs Ldlr^−/−^ and Ldlr^−/−^;Rag-2^−/−^. Each dot indicates results for an individual animal. (scale bar: 500 µm). **D.** Phenotype analysis of atherosclerotic plaques demonstrated more advanced lesion development in Socs-1^−/−^triple-KO (Socs-1^−/−^) mice. N = 5–8 mice/group.

The extent of atherosclerotic plaque development was analysed in complete aortas prepared en face and stained with Oil Red O and demonstrated significantly enhanced lipid depositions in vessels of Socs-1^−/−^triple-KO mice (figure 3A). Concomitantly, we also observed increased atherosclerotic lesion formation (figure 3B) and an accumulation of macrophages (MOMA-2 positive cells) in aortic roots of Socs-1^−/−^triple-KO mice (figure 3C). Atherosclerotic plaque progression was analysed using the recommendations of the report from the Committee on Vascular Lesions of the Council on Arteriosclerosis, American Heart Association (figure 3D). [20] Overall, very early lesions types dominated after 4 weeks of HCD regardless of the respective genotype. However, plaques obtained from Socs-1^−/−^ triple-KO mice proved to be the most advanced. To further characterize lesion composition we performed Sirius Red stainings but did not observe any significant extracellular matrix formation after 4 weeks of high-cholesterol dieting (figure S2A).

### SOCS-1 Regulates Monocyte Ly-6C^hi^ Subset Formation in Atherosclerosis-prone Mice

**Figure 4:**
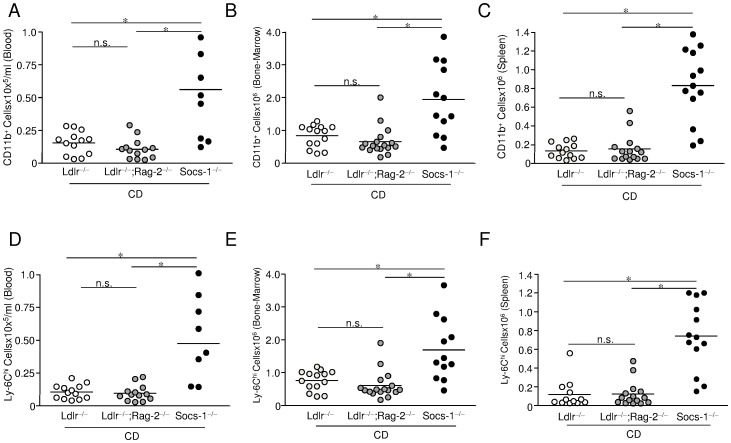
Impact SOCS-1 deficiency on monocyte formation under chow diet (CD). A-C. Peripheral blood (A), bone-marrow (B) and spleen (C) of age-matched mice from all three genotypes under CD were subjected to cell sorting. Monocytes were determined as CD11b^hi^ (CD90, B220, CD49b, NK1.1, Ly-6G)l^o^ cells. Triple-KO (Socs-1^−/−^) mice showed significantly enhanced numbers of circulating CD11b^+^ cells. **D-F.** Monocytosis was mainly due to an increase of the Ly-6C^hi^ subset. Horizontal bars represent mean. *p<0.05 vs Ldlr^−/−^;Rag-2^−/−^ and Ldlr^−/−^mice. Each dot represents the results for an individual animal of an independent experiment.

Cell sorting experiments were performed in age-matched mice after 4 weeks of chow diet (CD) or high-cholesterol diet (HCD). Despite the overall leukopenia of Socs-1^−/−^triple-KO mice under CD, flow cytometric analysis of blood samples (figure S1) demonstrated a marked increase in circulating CD11b^hi^ monocytes (figure 4A) that was mainly due to an increase of the Ly-6C^hi^ cell subset (figure 4D). Similar observations were made in bone-marrow (figure 4B and E) and spleens of Socs-1^−/^triple-KO mice (figure 4C and F). Analysis of the Ly-6C^lo^ subset revealed no differences between triple-KO mice and Ldlr^−/−^ mice, but significantly less circulating Ly-6C^lo^ cells in Ldlr^−/−^;Rag-2^−/−^ mice (data not shown).

**Figure 5:**
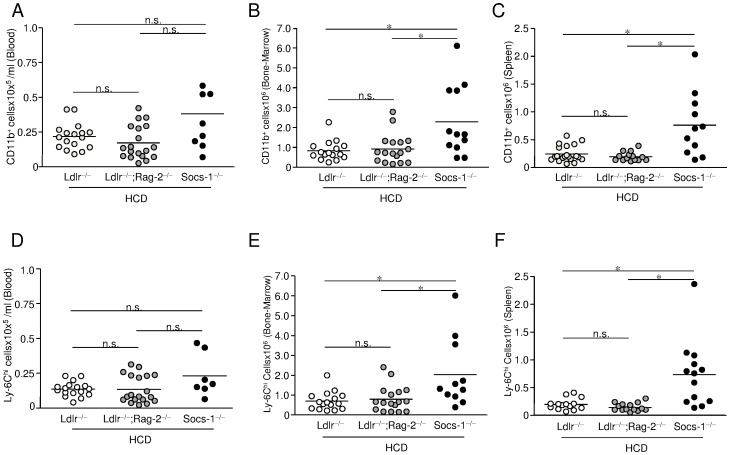
Impact SOCS-1 deficiency on monocyte formation under high-cholesterol diet (HCD). Peripheral blood, bone-marrow and spleen of age-matched mice from all three genotypes after 4 weeks of HCD were subjected to cell sorting. While cell numbers of CD11b^+^; Ly-6C^hi^ monocytes remained significantly higher in bone-marrow and spleen of Socs-1^−/−^ triple-KO (Socs-1^−/−^) mice, circulating CD11b^+^; Ly-6C^hi^ monocyte numbers decreased. Horizontal bars represent mean. *p<0.05 vs Ldlr^−/−^;Rag-2^−/−^ and Ldlr^−/−^ mice. Each dot represents the results for an individual animal of an independent experiment.

4 weeks of HCD resulted in a significant rise of total circulating leukocytes in Ldlr^−/^and Ldlr^−/−^;Rag-2^−/−^ mice compared to mice on CD (table 2). However, we observed a tendency toward decreased numbers of leukocytes and granulocytes in peripheral blood of Socs-1^−/−^triple-KO mice (table 2). Analysis of leukocyte subsets indicated a slight, but not statistically significant rise of CD11b^hi^ monocytes and Ly-6C^hi^ cells in peripheral blood of Ldlr^−/−^ and Ldlr^−/−^;Rag-2^−/−^ animals. In Socs-1^−/−^ triple-KO mice the proportion of circulating CD11b^hi^ monocytes and Ly-6C^hi^ cells decreased (figure 5A and D) while Ly-6C^hi^ monocytosis persisted in bone-marrow (figure 5B and E) and spleens (figure 5C and F). Numbers of Ly-6C^lo^ monocytes did not significantly change under HCD with regard to genotype or hematopoietic compartment (data not shown).

**Figure 6:**
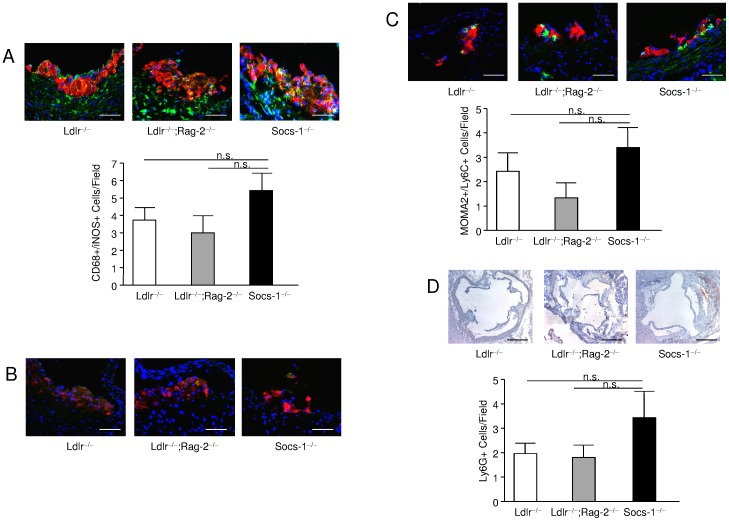
Impact of SOCS-1 deficiency on atherosclerotic plaque composition A. Atherosclerotic plaques from Socs-1^−/−^ triple-KO (Socs-1^−/−^) mice showed an increased CD68 (red);iNOS (green) double-positive cell content. **B.** CD68 (red); CD206 (green) double-positive cells were hardly detected. **C.** Atherosclerotic plaques from Socs-1^−/−^triple-KO (Socs-1^−/−^) mice contained slightly more MOMA-2 (red); Ly-6C (green) double-positive cells after 4 weeks of HCD as well as **D.** a higher number of Ly-6G positive cells (scale bar A–C: 20 µm, scale bar D:50 µm). N = 5–7 animals per group.

Considering the contribution of Ly-6C^hi^ monocytes to atherosclerotic plaque formation together with the sustained generation of these cells in bone-marrow and spleens of Socs-1^−/−^ triple-KO mice, we hypothesized, that the reduced number of circulating monocytes after 4 weeks of HCD might reflect an increased cell extravasation with subsequently enhanced lesion development at this particular point of time. Therefore, we performed double stainings of MOMA-2 and Ly-6C in atherosclerotic plaques obtained from Socs-1^−/−^ triple-KO mice. While the overall number of double-positive cells proved to be quite low and was constricted to small, very early lesions, these stainings indicated an enhanced number of MOMA-2/Ly-6C positive cells in Socs-1^−/−^triple-KO mice although these observations did not reach statistical significance (figure 6C). Given the neutrophilia in Socs-1^−/−^triple-KO mice, we also stained aortic roots for Ly-6G as a marker for neutrophils. Again, the total cell number detected was very low. However, quantitative analysis revealed increased lesion infiltration of Ly-6G positive cells in Socs-1^−/−^triple-KO mice (figure 6D).

### SOCS-1 Regulates Pro-atherogenic Macrophage Formation in Atherosclerosis-prone Mice

**Figure 7:**
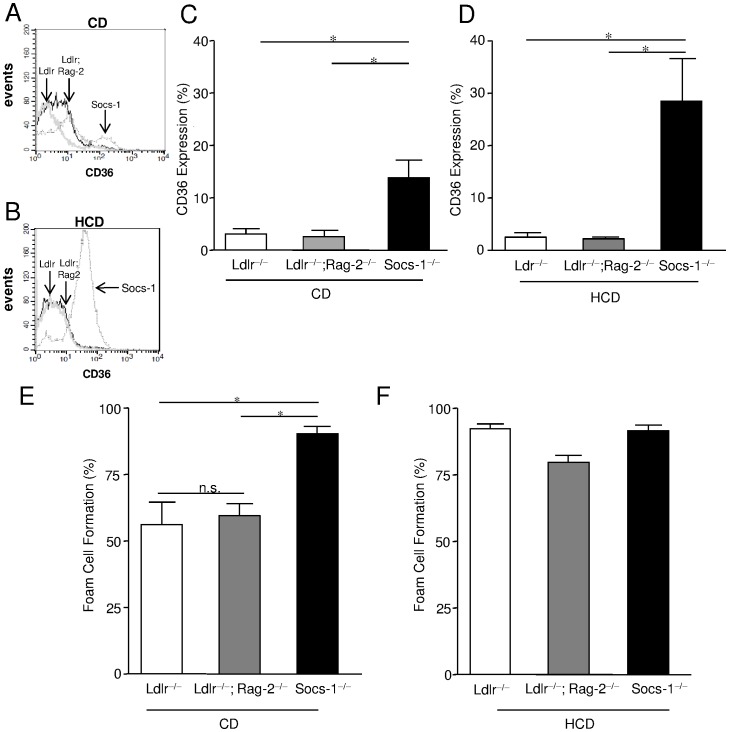
Impact of SOCS-1 deficiency on scavenger receptor CD36 expression and foam cell formation under chow diet (CD) and high-cholesterol diet (HCD). A and B. Representative histograms of CD36 expression of bone-marrow derived macrophages (BMDM) obtained from all 3 genotypes after 4 weeks of CD of HCD. **C and D.** BMDM of triple-KO mice (Socs-1^−/−^) displayed a significantly enhanced CD36 expression determined by flow cytometry under CD. Under HCD, CD36 expression further increased on Socs-1^−/−^ BMDM. **E.** BMDM from Socs-1^−/−^ mice showed significantly enhanced foam cell formation determined after stimulation with oxLDL after 4 weeks of CD. **F.** Under HCD foam cell formation did not differ between the genotypes anymore. Data are mean±SEM, *p<0.05 vs Ldlr^−/−^ and Ldlr^−/−^;Rag-2^−/−^, N = 5–13 mice/group.

To further investigate the impact of SOCS-1 on macrophage phenotype in atherogenesis, scavenger receptor (SR)-A and CD36 expression were determined on bone-marrow derived macrophages (BMDM) after CD and HCD. While SR-A expression did not differ between the genotypes (data not shown), macrophages received from Socs-1^−/−^triple-KO mice on CD already showed a significantly enhanced expression of CD36 compared to Ldlr^−/−^;Rag-2^−/−^ or Ldlr^−/−^ mice that further increased after HCD (figure 7A–D). Subsequently, stimulation with oxLDL resulted in a markedly higher proportion of foam cell formation in SOCS-1 deficient BMDM compared to BMDM derived from Ldlr^−/−^;Rag-2^−/−^ and Ldlr^−/−^ mice on chow diet that did not further increase after HCD (figure 7E–F). Given these findings, we also analysed intra-plaque macrophage phenotypes. In this regard, we performed double stainings using CD68 in combination with iNOS for detection of M1 macrophages (figure 6A) versus CD68 combined with CD206 for tracking of M2 macrophages (figure 6B). While we hardly detected any M2 macrophages regardless of the respective genotype, Socs-1^−/−^ triple-KO mice showed an enhanced proportion of M1 macrophages although it did not reach statistical significance due to the low overall cell number.

## Discussion

Atherosclerosis is a chronic inflammatory disease of the arterial cardiovascular system. [21] As SOCS-1 has been demonstrated to restrict the activation of various pro-atherogenic pathways, [2] we postulated that loss of SOCS-1 in an atherosclerosis-prone organism might aggravate plaque development.

To address this question, we generated Ldlr^−/−^;Rag-2^−/−^;Socs-1^−/−^triple-KO mice. These animals suffered from a complex systemic inflammation with familiar features of Rag-2^−/−^;Socs-1^−/−^ and Socs-1^−/−^ mice, including leucopenia and neutrophilia. Beyond that, the additional lack of the LDLR in Socs-1^−/−^ triple-KO mice resulted in a Ly-6C^hi^ cell-dependent monocytosis.

Ly-6C^hi^ monocytes are defined as a short-lived Cx_3_CR^lo^ cell subset that drives inflammation by extravasation, tissue infiltration and differentiation into phagocytes. In context with atherogenesis, they are considered as the major, pro-atherogenic monocyte population. [22] So far, data discussing the impact of SOCS-1 on monocyte generation or function has been limited to experimental settings of infection. [23] For example, human monocytes have been shown to up-regulate SOCS-1 in response to nitric oxide resulting in reduced secretion of IL-6 and IL-10. [24] In addition, increased SOCS-1 expression in monocytes infected with hepatitis C aggravates disease activity by modulating IL-12 secretion while in Ly-6C^hi^ monocytes isolated from bone-marrow of C57BL/6 mice infected with Listeria monocytogenesis early SOCS-1 expression is associated with host outcome. [25], [26].

As mentioned above, Ly-6C^hi^ monocytes are regarded as a vital source for plaque macrophages. These phagocytes undergo foam cell formation by up-take of oxidized LDL particles via scavenger receptors - a dominating event in early lesion development. Interestingly, macrophages derived from Socs-1^−/−^triple-KO mice on chow diet already displayed significantly enhanced scavenger receptor CD36 expression. Accordingly, we also found a profoundly increased foam cell formation of these cells ex vivo.

Spontaneous atherogenesis is scarce in Ldlr^−/−^ mice. Nevertheless, given the increased generation of Ly-6C^hi^ monocytes and activated macrophages together with the enhanced circulating levels of IL-6, TNF-α and MCP-1, we speculated that atherosclerotic plaque development might already occur in SOCS-1^−/−^triple-KO mice on chow diet. However, we did not observe lesion formation in the absence of hypercholesterolemia regardless of the genotype investigated.

Thus, we subjected Ldlr^−/−^, Ldlr^−/−^;Rag-2^−/−^ and Socs-1^−/−^ triple-KO animals to 4 weeks of HCD starting directly after weaning.

While lipoprotein fractions did not significantly differ between Ldlr^−/−^ and Socs-1^−/−^ deficient mice after the feeding period, they proved to be slightly lower in Ldlr^−/−^;Rag-2^−/−^ animals. These results are consistent with the observations of other groups investigating the impact of RAG-2 deficiency in the ApoE mouse model of atherosclerosis. Neither we nor previous studies have been able to elucidate the underlying mechanisms. However, regarding the importance of lymphocytes for barrier-functions – including the gastro-intestinal tract – one might speculate that lymphocyte depletion may disturb both, lipoprotein up-take as well as transport. [27], [28].

Despite the short period of HCD and the young age of animals, we found enhanced atherosclerotic lesion formation throughout the aorta as well as in aortic roots of Socs-1^−/−^triple-KO mice. Accordingly, investigation of plaque composition revealed an increased content of macrophages with pro-atherogenic M1 features in these mice. SOCS-1 is a known modulator of macrophage activation by suppressing CD40, IL-6 and TNF-α expression. [29], [30] Furthermore, as shown by Whyte et al. in context with parasite infection, SOCS-1 may confine classically activated M1 macrophage formation and foster alternatively activated - potentially anti-atherogenic - M2 macrophage generation. [3] Thus, these observations support the pro-atherogenic impact of SOCS-1 deficiency in this mouse model. The fact that we hardly detected any M2 macrophages regardless of the genotype examined may reflect the mainly pro-inflammatory sub-intimal environment at this particular time of plaque development. [31].

Overall, lesion phenotype analysis confirmed that atherogenesis proved to be the most advanced in Socs-1^−/−^triple-KO mice.

Flow cytometric analysis of peripheral blood demonstrated markedly decreased numbers of CD11b^+^;Ly-6C^hi^ monocytes in Socs-1^−/−^ triple-KO mice after 4 weeks of HCD, while these cells remained increased in bone-marrow and spleen. We hypothesized, that the reduced number of circulating monocytes in Socs-1^−/−^triple-KO mice might have reflected an increase in vascular extravasation. [29] We resigned from performing sorting experiments with SOCS-1 deficient aortic tissue due to the short feeding period, the young age of mice and the overall low circulating CD11b^+^;Ly-6C^hi^ cell number. Instead, we stained aortic roots for MOMA-2, Ly-6C double-positive cells. Although the fate of Ly-6C^hi^ monocytes after entering the sub-intimal space likely depends on the local cytokine/chemokine balance at the particular time of entrance and thus, may change during plaque development, we detected more MOMA-2, Ly-6C double-positive cells in developing Socs-1^−/−^triple-KO lesions.

Given the systemic neutrophilia observed in SOCS-1 deficient animals, we also examined atherosclerotic plaques for neutrophil infiltration. Indeed, we found more Ly-6G positive cells in lesions derived from Socs-1^−/−^triple-KO mice. Neutrophils have recently gained attention and may particularly contribute to early atherogenesis by production of oxygen radicals and secretion of pro-inflammatory granules. [32] Therefore, neutrophils may be involved in the atherogenic phenotype of SOCS-1 deficient mice in this study.

We are also aware of the fact, that disturbed T- and B-cell development caused by loss of RAG-2 might have influenced atherogenesis in this mouse model especially since RAG-2 deficiency obviously modulated lipoprotein homeostasis. In this context Reardon et al found a close correlation between lipoprotein levels and lesion extent in Rag-2^−/−^;ApoE^−/−^ mice as well as a site-specific difference in plaque development. However, others did not detect any obvious effects of RAG-2 on lesion size. [27], [28] In the study presented, we did not observe significant differences in plaque development between Ldlr^−/−^ and Ldl^−/−^;Rag-2^−/−^ mice despite a tendency towards more advanced lesions in Ldlr^−/−^;Rag-2^−/−^ animals. These results surely do not negate the impact of lymphocytes on atherogenesis. Instead they may rather be a consequence of this specific study design with very young mice undergoing a short feeding period.

One might also argue that the atherogenic phenotype of Socs-1^−/−^triple-KO animals may be secondary to the ongoing systemic inflammation and therefore, of constricted biological relevance. However, we did not find atherosclerotic plaque formation in Socs-1^−/−^ triple-KO mice until induction of hypercholesterolemia despite Ly-6C^hi^ monocytosis, activation of macrophages and neutrophilia under chow diet conditions indicating that a specific, pro-atherogenic trigger was necessary for induction of plaque development. Furthermore, deletion of a single SOCS-1 allele resulted in effects similar to those observed in Socs-1^−/−^triple-KO animals (figure S2B–D). In summary, this study demonstrates the athero-protective nature of SOCS-1 in experimental, murine atherosclerosis.

## Supporting Information

Figure S1 **Cell sorting of blood derived from Ldlr** ^−/−^ **, Ldlr** ^−/−^ **;Rag-2** ^−/−^ **and Ldlr** ^−/−^ **;Rag-2** ^−/−^ **;Socs-1** ^−/−^ **. A and B.** Representative dot plots showing cell sorting of CD11b^hi^ (CD90, B220, CD49b, NK1.1, Ly-6G)^lo^ cells from blood derived from all three, age-matched genotypes after (**A**) 4 weeks of chow diet (CD) or (**B**) 4 weeks of high-cholesterol diet (HCD). **C and D.** Representative histograms demonstrating the proportion of Ly-6C^hi^ blood monocytes among CD11b^hi^ (CD90, B220, CD49b, NK1.1, Ly-6G)^lo^ cells in all three genotypes. Independent cell sorting experiments were performed in specimens derived from 8–17 animals per group.
(TIF)

Figure S2 **Impact of SOCS-1 on extracellular matrix formation and Impact of SOCS-1^+/−^ on atherosclerotic plaque development and plaque content of macrophages after 4 weeks of high-cholesterol diet (HCD). A.** Representative pictures demonstrating that atherosclerotic plaque development did not involve significant collagen formation after 4 weeks of HCD regardless of the genotype investigated **B.** Aortas derived from Socs-1^+/−^ mice showed significantly enhanced lipid depositions after en face preparation and staining with Oil Red O after 4 weeks of HCD. Results were comparable to those derived from Socs-1^−/−^triple-KO mice **C and D**. Aortic roots of Socs-1^+/−^ mice also displayed increased macrophage content after staining with MOMA-2 (**C**) and enhanced lipid depositions after staining with Oil Red O (**D**) after 4 weeks of HCD. Both results were comparable to those derived from Socs-1^−/−^ triple-KO mice. Horizontal bars represent mean, *p<0.05 vs Ldlr^−/−^ and Ldlr^−/−^;Rag-2^−/−^ (**C:** scale bar: 500 µm, **D:** scale bar: 50 µm). Each dot indicates results for an individual animal.
(TIF)
