# Supplementary figures and images for: Role of Suppressor of Cytokine Signaling-1 In Murine Atherosclerosis
Source: PLoS One. 2012 Dec 27;7(12):e51608. doi: 10.1371/journal.pone.0051608 (PMC3531439; doi:10.1371/journal.pone.0051608)

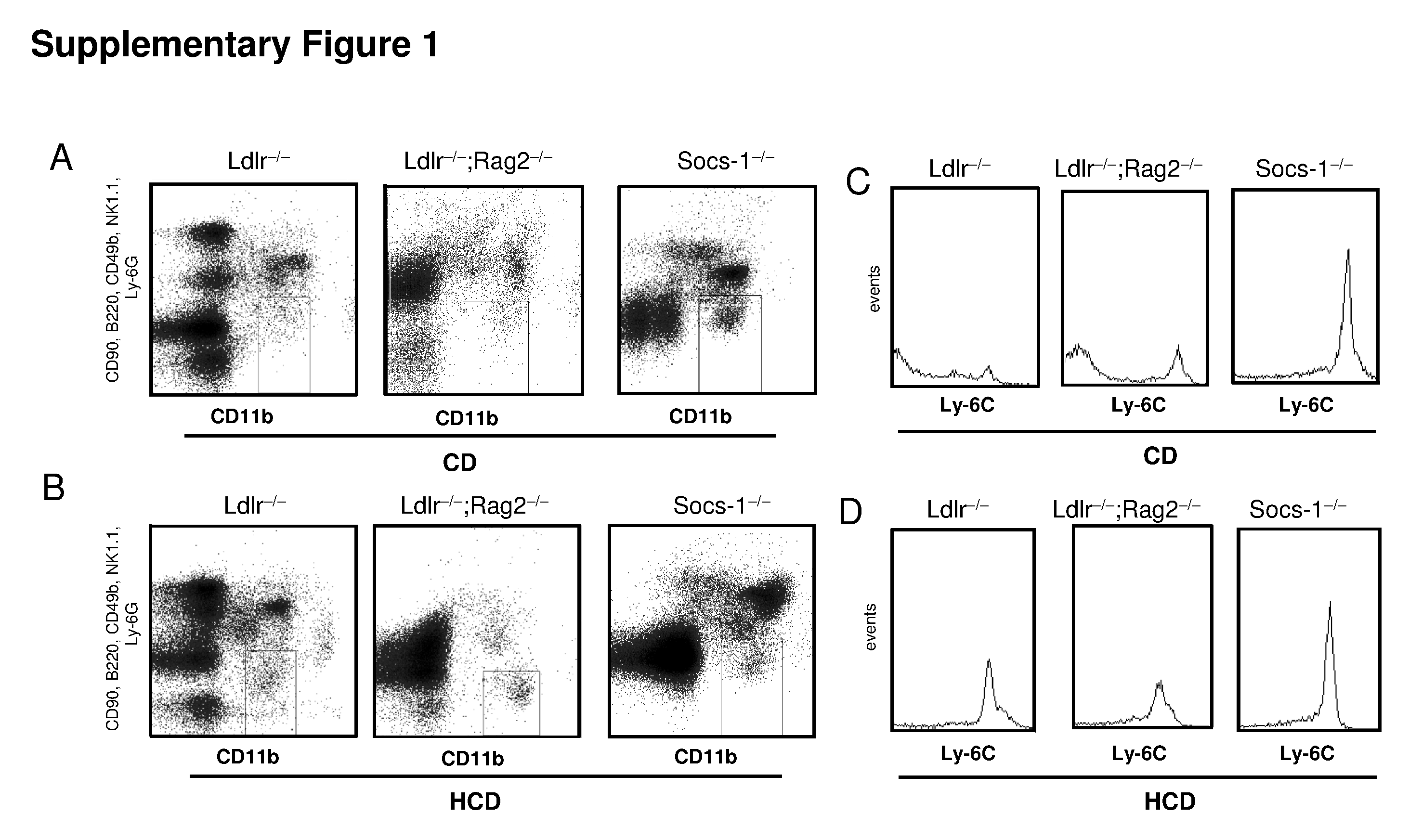

Supplement: Figure S1 — Cell sorting of blood derived from Ldlr −/− , Ldlr −/− ;Rag-2 −/− and Ldlr −/− ;Rag-2 −/− ;Socs-1 −/− . A and B. Representative dot plots showing cell sorting of CD11bhi (CD90, B220, CD49b, NK1.1, Ly-6G)lo cells from blood derived from all three, age-matched genotypes after (A) 4 weeks of chow diet (CD) or (B) 4 weeks of high-cholesterol diet (HCD). C and D. Representative histograms demonstrating the proportion of Ly-6Chi blood monocytes among CD11bhi (CD90, B220, CD49b, NK1.1, Ly-6G)lo cells in all three genotypes. Independent cell sorting experiments were performed in specimens derived from 8–17 animals per group. (TIF) [file pone.0051608.s001.tif]

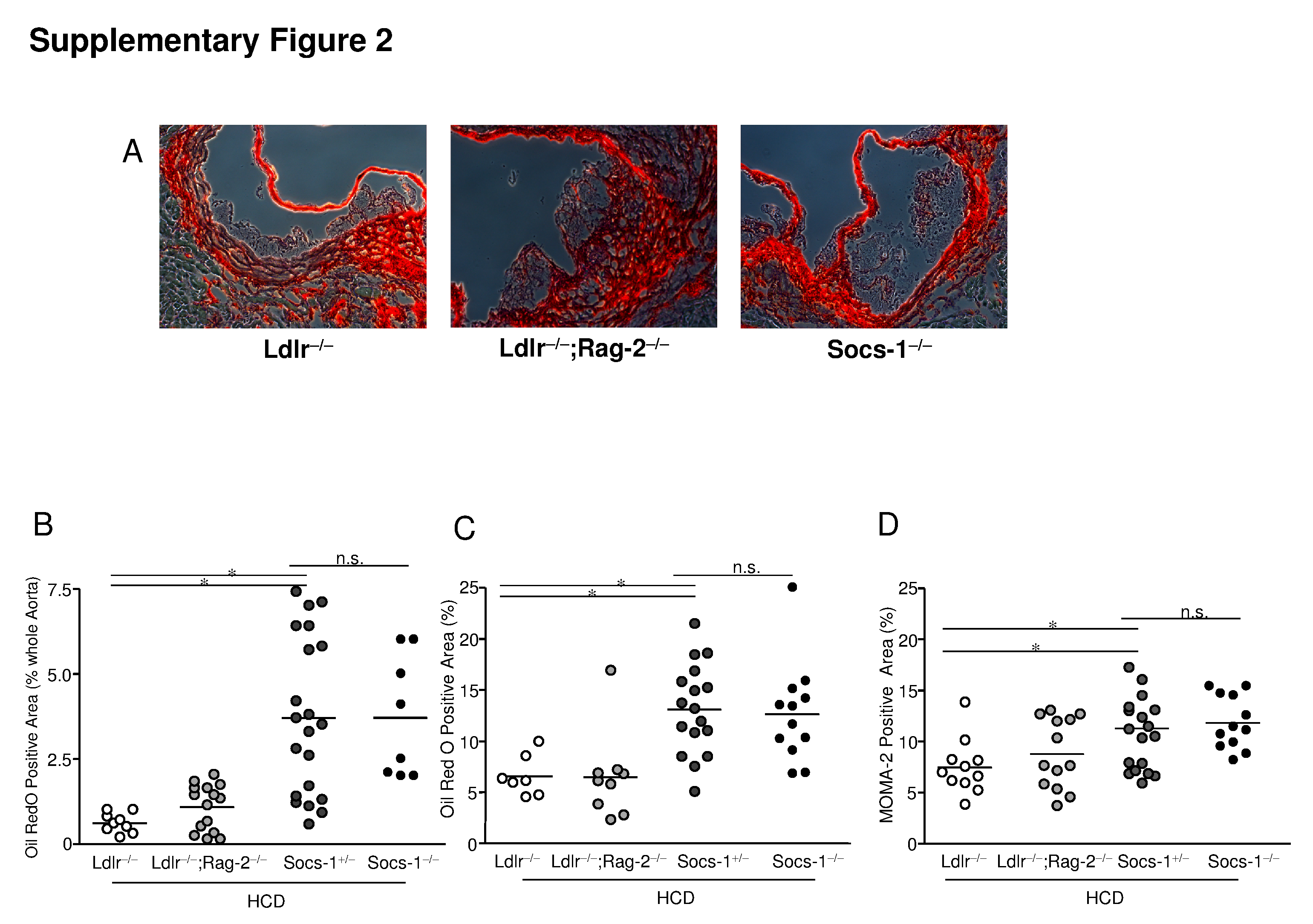

Supplement: Figure S2 — Impact of SOCS-1 on extracellular matrix formation and Impact of SOCS-1+/− on atherosclerotic plaque development and plaque content of macrophages after 4 weeks of high-cholesterol diet (HCD). A. Representative pictures demonstrating that atherosclerotic plaque development did not involve significant collagen formation after 4 weeks of HCD regardless of the genotype investigated B. Aortas derived from Socs-1+/− mice showed significantly enhanced lipid depositions after en face preparation and staining with Oil Red O after 4 weeks of HCD. Results were comparable to those derived from Socs-1−/−triple-KO mice C and D. Aortic roots of Socs-1+/− mice also displayed increased macrophage content after staining with MOMA-2 (C) and enhanced lipid depositions after staining with Oil Red O (D) after 4 weeks of HCD. Both results were comparable to those derived from Socs-1−/− triple-KO mice. Horizontal bars represent mean, *p<0.05 vs Ldlr−/− and Ldlr−/−;Rag-2−/− (C: scale bar: 500 µm, D: scale bar: 50 µm). Each dot indicates results for an individual animal. (TIF) [file pone.0051608.s002.tif]
